# The ‘ideal’ dancer: An investigation into predictors of body image dissatisfaction among male dancers, female dancers and their non-dancing counterparts

**DOI:** 10.1371/journal.pone.0313142

**Published:** 2024-11-07

**Authors:** Jessica E. Boyes, Katri K. Cornelissen

**Affiliations:** Department of Psychology, Northumbria University, Newcastle Upon Tyne, United Kingdom; Novi Sad School of Business, SERBIA

## Abstract

Body image dissatisfaction is consistently highlighted as a precursor for eating disorders, arising from several factors. One factor surrounds social expectations of being thin, which can be emphasised in certain physical activities, like dance. Whilst research is available for body image dissatisfaction among female dancers, literature has neglected the male demographic. The present study investigated seven predictors of body image dissatisfaction within male and female dancers, from a variety of genres. The study employed a non-experimental quantitative method, utilising an independent groups correlational design. The role of clothing practices, body ideal internalisation and sociocultural influences were investigated as predictors for body image dissatisfaction. Data from 180 participants (mean age = 23.7, SD = 7.51) was analysed from an online survey. Hierarchical multiple regression revealed that the model consisting of all predictors significantly accounted for over 60% of variance in body image dissatisfaction scores within all independent groups. The individual contribution of predictors differed between groups, with clothing practice being the only significant predictor in all four groups. Media pressure was a significant predictor within non-dancer groups only. This research highlights the importance of clothing practices, specifically tight-fitting garments within the dance environment and general population. Future research should investigate potential differences in the predictors between each level of dance.

## 1.Introduction

Body image is a multidimensional construct relating to the cognitions, perceptions and emotions one has about their body. Distorted body image refers to a disturbed perception of how one views their body and is often seen in eating disorders (ED) like Anorexia Nervosa [1,2]. Whilst body image comprises two established components [3], research has mostly focused on the attitudinal component due to evidence linking it with ED development and other maladaptive outcomes like mood disorders [4,5]. The attitudinal component captures the subjective opinion of one’s physical self, specifically relating to shape, weight and height among other aspects [6] and is often assessed in terms of body dis/satisfaction.

Body image dissatisfaction (BID) emergence is often explained using the Tripartite Influence Model (TIM) proposed by Thompson et al. [7]. TIM proposes that BID emerges in response to social pressures from media, family and peers which is mediated by social comparison and societal ideals that impose insurmountable standards for bodily appearance [8]. Individuals who internalise dominant beliefs and ideals surrounding beauty standards promoted within their socio-cultural environment, whilst simultaneously feeling they do not satisfy the standard, are at an elevated risk of experiencing BID [9]. Modern society favours a slender body, especially for westernised females, resulting in internalisation of a thin ideal which is associated with BID [10,11]. Among males, internalisation of a muscular ideal is typically associated with BID [12] although, other research suggests male thin ideal internalisation is positively related to disordered eating [13]. More recent evidence highlights male concerns surrounding thinness whilst simultaneously wishing to develop muscularity [14,15], referring perhaps to the athletic, paradoxical body ideal. In much of the literature, the muscular and athletic ideal are viewed as one, despite some evidence revealing their individual influences [16]. This may be a result of the predominant measure (Social Attitudes Towards Appearance Questionnaire-4) including one subscale measuring athletic/muscular internalisation together [17].

TIM elements appear central within the dance community and are arguably facilitated by the environment dance affords [18]. Dance is considered an aesthetic discipline [19,20] offering opportunities across all ages, abilities and levels. Individuals involved in dance typically present with greater weight preoccupations, BID and disordered eating behaviours compared to the general population [21]. Meta-analyses and systematic review data suggest that there are higher levels of BID and disordered eatings tendencies in the dance population as compared to control population [22–24]. The heightened body related issues dancers face is suggested to result from the ruthless nature of the dance world [25,26]. There is though some evidence suggesting that dance participation promotes positive well-being and body image [27,28]. A likely explanation for contradictory findings may surround differences between dance types and abilities [29]. For instance, high disordered eating rates and BID have been consistently reported among professional ballet dancers [21,30]. It indeed is generally common, particularly at the elite level, that there is a pressure to maintain a thin, “balletic” physique [31–33]. Ballerinas are therefore seen as a risk group for suffering from BID and ED, possibly in consequence of the environmental facilitation of tripartite influences [22,34]. In contrast, other forms of dance may also be subject to strong thin ideal internalisation pressures, for example, like ballet, jazz dancers are known for their lean physique [35].

Alongside tripartite influences, research has suggested that the clothing used, particularly within ballet practise, acts as a risk factor for BID [36] Qualitative research has demonstrated feelings of unease toward one’s body, among samples of female dancers, due to strict implementation of fitted clothing that highlights the body’s silhouette [18,36]. Such findings consolidate older reports of the strict use of leotards and tights, paired with bodily obsession, that seems to pose significant issues on wellbeing and impacts dancer performance [37]. To date, male dancers and dancers of other genres have not been represented in the samples, with studies frequently focusing on female ballet dancers at pre-professional and professional level.

Most, if not all research discussed above has focused solely on ballet. As one of many dance disciplines, focusing solely on ballet results in a narrow understanding of BID and predictors of body dissatisfaction in the given population. Investigation utilising additional genres (e.g., tap, contemporary, jazz and hip hop) is equally important to develop the full understanding of BID [38]. some existing research has touched on contemporary dance, this research has been in relation to fitness rather than body image [39]. Aside that, street dance has been briefly explored, with focus on body appreciation [40].

Furthermore, literature has mainly focused on professional dancers neglecting many other levels [27,41]. Ravaldi et al. [25] reported high levels of BID and inappropriate eating behaviours/attitudes among amateur dancers, suggesting the pressures placed upon professionals may also pose an influence on non-professional dancers. Therefore, when investigating contributing factors of BID, it would be beneficial to incorporate various dance abilities and styles, enabling a wider representation which current research lacks.

The BID and dance field also appears to lack a comprehensive evidence base using male participants. There appears to be an overreliance on females within BID research, limiting implications for the male demographic [42]. The limited available research utilising male dancers reports that males view themselves as being different from non-dancers [43] and typically report lower BID compared to females [44]. However, that does not imply male dancers are immune to social pressures from the dance world. Given the association between disordered eating and BID within female dancers, investigations of these concerns and what may predict them among males is vital to prevent the notion that BID is a female-oriented problem [45].

This research will explore BID concerns inclusive of male dancers, various dance styles and levels. Moreover, the current research will investigate several predictors, including clothing practices, and thus not limiting to TIM elements, typically researched within the literature [46]. Furthermore, the sociocultural aspect central to TIM is often investigated as one concept, even though research has evidenced that the different components of TIM can have an independent influence on bodily concerns [47]. Hence, this study will explore the influence of family, peers and media individually, to gain a more precise understanding of TIM factors that can predict BID among both male and female dancers. The research question is “Which factors predict body dissatisfaction among male dancers and does this differ to female dancers as well as those not involved in dance?”

This study is set to investigate seven factors that may predict BID among a sample of dancers and non-dancers, both male and female. The seven factors are the thin, athletic and muscular ideal alongside clothing practices, family, peers, and media pressures. The current research is testing the following hypotheses:

H1: Greater media pressure will significantly predict higher BID over and above other factors, within both male and female non-dancers. Media pressures are one of the main predictors of BID in the general female population [7,34] and similar results have been evidenced among males [48].
H2: Greater levels of discomfort in tight-fitting clothing will significantly predict higher BID scores for female dancers, above other factors. BID is associated with clothing practices, particularly tight-fitting clothes, within female dancers [18,37].
H3: Greater internalisation of the athletic ideal will significantly predict higher BID scores within male dancers, above other factors. Despite little research investigating athletic and muscular ideals independently from one another, acute exposure to athletic ideal increased self-reported BID among males, whereas muscular ideal exposure did not [16].
H4: The remaining factors will follow a similar trajectory, whereby greater internalisation/pressures will significantly predict higher BID.

## 2. Method

Ethical approval for the study procedures and methods was granted by Northumbria University Psychology Department Research Ethics Committee (reference number: 0514). Written informed consent was gained from participants prior to study participation.

### 2.1 Participants

Prior to recruitment, a prospective power analysis was conducted using G*Power 3.1 [49]. Among sociocultural influences on BID literature, effect sizes, in line with Cohen’s f^2^, ranged from small to medium (.07 to .25) averaging at .13 [50–52]. Therefore, an effect size of .13 and a power of .8, to reduce type II error, was used [50]. G*Power proposed minimum sample size to be 118 participants, approximately 30 participants per independent group.

Recruitment ran from the 4^th^ of November 2022 to the 5^th^ of December 2022. Opportunity to participate was created globally, though due to recruitment channels used, it is likely the participants were inevitably based in the UK. Participants had to be biologically male or female, identify as their gender assigned at birth and over 18, to expand the adolescent sample research that is previously most reported (34,53]. Individuals diagnosed with an ED, at the time of study, were excluded from survey completion to prevent psychological harm and adhere to the research’s subclinical nature. Participants were recruited to an opportunistic sample via social media advertisement (e.g., Facebook). Additionally, personal contacts were utilised for recruitment, ensuring the proposed sample size was met.

Participants were first asked to give demographic information, including age, weight, height and biological sex. Dance specific demographics were asked such as, dance ability, genres participated in, dance involvement in years, hours per week and hours coached per week. When selecting genres involved in, participants had the option to select multiple responses including ‘other,’ allowing for open-ended responses not covered by the multiple-choice list. Dance ability was operationalised into four categories (non-dancer, amateur, pre-professional, professional) and presented as multiple-choice requiring one answer. Non-dancer was described as an individual with no past or present involvement in dance (e.g., involvement refers to 1 year minimum). Amateur was described as an individual with an involvement in dance that did not reach a level of formal training that would’ve resulted in professional qualification (e.g., dance training at a local dance school rather than a dance college or conservatoire). Pre-professional was described as an individual in receipt of formal training, pursuing a career within dance, but not yet reached professional qualification (e.g., currently undertaking a dance course at an institution such as Laine Theatre Arts etc). Professional was described as an individual who holds formal dance qualifications and has a career within the dance world (e.g., working within theatre, cruise ships, dance teacher etc). Participant characteristics are reported below (Table 1).

**Table 1:** Participant characteristics of dancers and non-dancers.

			Dancers (N = 107)					Non-Dancers (N = 73)		
	Male (N = 34)			Female (N = 73)					Male (N = 38)	Female (N = 35)	
		Mean (SD)			Mean (SD)				Mean (SD)	Mean (SD)	
**Age**		21.94 (4.00)			20.73 (3.30)				27.66 (9.78)	27.31 (10.06)	
**Weight (Kilograms)** **Height (Meters)**		72.00 (6.57) 1.78 (0.06)			61.87 (16.82) 1.63 (0.07)				85.55 (17.26) 1.80 (0.10)	67.04 (14.99) 1.62 (0.10)	
**Body Mass Index (BMI)** **Involvement in years** **Hours per week** **Hours coached per week**		22.80 (2.69) 9.03 (4.94) 19.50(17.45) 11.03 (9.97)			23.24 (6.57) 14.79 (5.40) 7.49 (7.51) 5.98 (6.70)				26.52 (5.25) 0 0 0	25.51 (5.60) 0 0 0	

The whole cohort comprised 314 participants, whereby 134 were removed due to incomplete data (n = 101), exclusion criteria violation (n = 19), lack of consent (n = 13) and ambiguous self-reported weight (n = 1). Therefore, attrition rate was 42.7%. Subsequently, 180 consenting participants remained for data analysis aged between 18 and 58 (Mean = 23.7, SD = 7.51) of which, 108 were female (Mean Age = 22.86, SD = 7.00) and 72 were male (Mean Age = 24.96, SD = 8.10).

Statistical testing was carried out to comprehensively understand the participant group, pertaining to BMI and age. A Shapiro-Wilk test of normality was conducted, revealing BMI and age across the four groups to violate normal distribution. Therefore, the non-parametric independent samples Kruskal-Wallis test was used. The test revealed that BMI significantly differed across the groups, χ^2^(3, N = 180) = 26.132, p < .001, as did age, χ^2^(3, N = 180) = 47.967, p < .001. Multiple pairwise comparisons, with a Bonferroni corrected significance level, revealed significant differences in BMI between female dancers (M = 23.24, SD = 6.57) compared to female non-dancers (M = 25.51, SD = 5.60) p < .027, and male non-dancers (M = 26.52, SD = 5.25) p < .001. A significant difference was also revealed between male dancers (M = 22.80, SD = 2.69) and male non-dancers (M = 26.52, SD = 5.25) p < .001. Regarding participant age, multiple pairwise comparisons revealed a significant difference between female dancers (M = 20.73, SD = 3.30) compared to female non-dancers (M = 27.31, SD = 10.06) p < .001 and male non-dancers (M = 27.66, SD = 9.78) p < .001. Significant differences were revealed between male dancers (M = 21.94, SD = 4.00) compared to female non-dancers (M = 27.31, SD = 10.06) p = .008 and male non-dancers (M = 27.66, SD = 9.78) p = .002.

Percentage of dancers in each level was calculated. Males (n = 34): amateurs (29.4%), pre-professionals (38.2%), professionals (32.4%). Females (n = 73): amateurs (64.4%), pre-professionals (31.5%) and professionals (4.1%). Due to a lack of variance, no significance testing was conducted; it was deemed appropriate to describe this at participant level. A noticeable difference existed between male and female professional dancer percentages. Similarly, the percentage of female amateurs was much larger than male amateurs, whilst the percentage of pre-professionals remains similar across both sexes. It is noted that there was proportionally a lower number of female professionals compared to male professionals. An explanation for such a difference could surround the volunteer sampling, whereby male professionals were more eager to participate; the primary aim of this research was to increase representation of the male dancer demographic within body image literature.

Regarding genres trained in, dancers were able to select multiple answers. Overall, 94.4% of the dancers selected more than one genre and 78.3% selected other, entering styles such as ballroom, salsa and lyrical. 47.2% of females selected that they had ballet training compared to 29.2% of males. Among males a higher percentage of participants had training in hip hop (33.3%) than in ballet (29.2%). Within female dancers, a slightly higher percentage of participants had training in jazz (53.7%) than in ballet (47.2%). Thus, the sample may be more representative of other genres compared to literature in this field.

### 2.2 Materials

#### i. Body Shape Questionnaire-16B (BSQ-16B)

The BSQ-16B is a self-administered scale, assessing bodily shape and size concerns [53]. Initially, Cooper et al. [54] proposed a 34-item BSQ frequently recommended for use in clinical ED settings [55]. Although the 34-item BSQ demonstrated sound psychometric properties, it has faced critiqued for being unnecessarily long given the unidimensional nature of the items [56,57]. Research suggests shorter questionnaires receive higher response rates [58,59] highlighting the revised BSQ-16B as more appropriate for the research. Items are rated along a 6-point Likert scale from 1 (never) to 6 (always) and consist of items such as, ‘Have you felt excessively large and round?**’** A total is produced by adding the 16 item scores, with the minimum score 16 indicating little to no signs of BID compared to the maximum score 96 indicating high BID. The BSQ-16B has demonstrated excellent reliability and in this study Cronbach’s Alpha was .952 across all participants, consistent with previous reports (Cronbach’s Alpha ranging from a = .94 to .95) [56,60].

#### ii. Sociocultural Attitudes Towards Appearance Questionnaire -4 (SATAQ-4)

The 22-item SATAQ-4 employs a five-factor structure to assess internalisation of appearance ideals and related pressures building from TIM [7,17]. Inclusion of thin and muscular appearance ideals within SATAQ-4 addressed conceptual limitations highlighted in previous versions, therefore, proving most appropriate for this study [17]. The study will employ SATAQ-4 except for the muscular/athletic subscale; in-house designed questions aligning with the research aims have been produced addressed muscular and athletic internalisation separately. Items are rated on a 5-point Likert scale from 1 (definitely disagree) to 5 (definitely agree). An example item is, ‘I think a lot about looking thin.’ As this research is investigating TIM independently, item scores are added per subscale to produce a total. The minimum score for thin subscale is 5 and for other subscales is 4. The maximum score is 25 for thin subscale and 20 for other subscales, scoring highly is indicative of greater internalisation and societal ideal pressures. SATAQ-4 has excellent reported psychometric properties; each subscale producing Cronbach’s Alpha ranging from .83 to .93 [17]. Cronbach’s Alpha calculated in this study produced alpha values of .80, .92, .88 and .92 for thin ideal, family, peer and media pressures respectively.

#### iii. In house published questions

15 in-house questions were produced to measure clothing practices, athletic and muscular ideal internalisation (five questions per predictor). Although ideal internalisation is measured by SATAQ-4, items do not clearly distinguish between the athletic and muscular ideal; recent findings suggest these ideals should viewed independently (Robinson et al., 2017) for a more comprehensive understanding of BID. In-house questions were created from literature highlighting distinct themes within muscularity and athleticism internalisation [13,61]. An example item for the athletic ideal is ‘I aspire to look slim and toned,’ and for the muscular ideal is ‘I aspire to “bulk up” and look muscular.’ The Body Image Avoidance Questionnaire [62] with reported Cronbach’s alpha of .89 was utilised alongside other research [63] to create clothing practice questions. An example item is ‘I wear baggy clothing during physical activity.’ One item was reverse scored to reduce acquiescence bias [64]. Items were scored on a 5-point Likert scale from 1 (definitely disagree) to 5 (definitely agree). All item scores were added to create a total for each of the three scales, the minimum score per scale was 5 and the maximum score was 25, higher scores indicated greater internalisation of ideals and discomfort in tight clothing. Reliability was calculated revealing Cronbach’s Alpha values of .81, .90 and .86 for athletic ideal, muscular ideal and clothing practices respectively.

### 2.3 Procedure

An anonymous link led participants to the information sheet which outlined the study, stated the possibility of slight psychological discomfort and provided helplines alongside researcher contact information. Participants were reminded of their freedom to withdraw at any time during survey completion and up to one month afterwards. Confidentiality was addressed; participants were reassured their data would remain anonymous throughout. Participants gave written informed consent before giving a code word to identify their data, should they want to withdraw. Participants were then able to complete the survey, starting with demographic information progressing onto BSQ-16B, SATAQ-4 and in-house created questions. Following successful completion, participants were presented with a debrief which reinstated investigation purposes, researcher contact information, helplines and data withdrawal. Overall, procedure completion took approximately 15 minutes.

### 2.4 Statistical procedure

Prior to any analysis several participants were removed for reasons such as, missing data and exclusion criteria violation. The non-parametric independent groups Kruskal Wallis test and pairwise comparisons compared group differences for participant characteristics (BMI and age); due to violation of normal distribution. Subsequently, it became apparent that one male dancer participant presented as a potential outlier; all dance hours were reported as zero, therefore, it was deemed appropriate to remove his data at this stage before further analyses, resulting in the final cohort as n = 179. Normal distribution was assumed, using Q-Q plots. Univariate analyses utilised one-way independent ANOVA to reveal significant differences between the total predictor and outcome variable scores of each group. Sub-analysis of BID within the dancer population was completed, whereby a non-parametric Kruskal-Wallis test and pairwise comparisons were utilised following normality testing. Independent samples t-test was used to compared BID between dancers with ballet training against dancers from other genres. A bivariate correlation matrix was produced to assess the variable relationships, ensuring no singularity or multicollinearity. As all relevant assumptions were met, hierarchical multiple linear regression was carried out four times, for each independent group. For all groups, age and BMI were entered into block one as a control due do their correlation with one another. For all groups, block two consisted of age, BMI and the seven predictors (thin ideal, athletic ideal, muscular ideal, clothing, family, media and peer pressures). The change in R^2^ was assessed to investigate the variance significantly explained by block two. Finally, individual predictor contribution was determined, enabling researchers to assess which predictor/s significantly contributed to the predictor model.

## 3. Results

### 3.1 Treatment of data

Qualtrics data was exported into SPSS (version 28) whereby all descriptive and inferential statistical analyses was carried out. Prior to conducting data analyses, item 3 from the clothing sub section was reverse scored in SPSS. Total scores were calculated, per participant, for each of the predictors. Box plots were then produced prior to univariate analyses using the participant questionnaire scores per independent group, to highlight potential outliers. No concerning outliers were identified.

### 3.2 Univariate analyses

A mean score per independent group, for each predictor variable and the outcome variable was calculated.

Following ANOVA revealing significant differences between groups (Table 2), Tukeys HSD was implemented for post hoc testing. Regarding BID, male non-dancers scored significantly lower (M = 42.63, SD = 17.70) than female non-dancers (M = 55.17, SD = 18.97), p = .010, and female dancers (M = 51.36, SD = 16.07), p = .050. Regarding thin ideal internalisation, both female dancers (M = 18.33, SD = 3.75), p < .001, and female non-dancers (M = 17.11, SD = 5.03), p = .023, had significantly higher internalisation compared to male non-dancers (M = 14.39, SD = 3.95). Regarding media pressure internalisation, female non-dancers (M = 16.63, SD = 3.33) reported significantly higher internalisation than male non-dancers (M = 12.76, SD = 4.46), p < .001, and male dancers (M = 13.00, SD = 4.37), p = .003. Similarly, female dancers (M = 15.89, SD = 4.32) reported higher internalisation than male non-dancers (M = 12.76, SD = 4.46), p = .001, and male dancers (M = 13.00, SD = 4.37), p = .007. Tukeys HSD revealed that for muscular ideal internalisation, female non-dancers (M = 11.77, SD = 4.62) reported significantly lower internalisation compared to male non-dancers (M = 15.21, SD = 5.74), p = .014, male dancers (M = 17.24,SD = 4.91), p < .001, and female dancers (M = 14.47,SD = 4.34), p = .036. Similarly, female dancers (M = 14.47,SD = 4.34) scored significantly lower compared to male dancers (M = 17.24,SD = 4.91), p = .034. For athletic ideal internalisation, male non-dancers (M = 15.89, SD = 4.48) reported significantly lower internalisation than male dancers (M = 20.88,SD = 2.91), p < .001, and female dancers (M = 19.23,SD = 2.92), p < .001. Similarly, female non-dancers (M = 16.26, SD = 3.93) scored significantly lower for athletic ideal internalisation compared to female dancers (M = 19.23,SD = 2.92), p < .001, and male dancers (M = 20.88,SD = 2.91), p < .001. Finally, for clothing related pressures, female non-dancers (M = 18.60, SD = 4.92) reported significantly higher pressures compared to male non-dancers (M = 14.97, SD = 5.36),p = .010, male dancers (M = 13.61, SD = 4.77), p < .001, and female dancers (M = 15.41, SD = 4.69), p = .010. No other significant differences were found.

**Table 2:** Mean and standard deviation scores for predictor variables and outcome variable (body image dissatisfaction) in full cohort (n = 179).

		Dancers (N = 106)				Non-Dancers (N = 73)				
	Male (N = 33)		Female (N = 73)				Male (N = 38)	Female (N = 35)			
	Mean (SD)		Mean (SD)				Mean (SD)	Mean (SD)		p	
**Body Image Dissatisfaction**	45.82 (16.13)		51.36 (16.07)				42.63 (17.70)	55.17 (18.97)		.007	
**Thin Ideal** **Family** **Peers** **Media** **Muscular Ideal** **Athletic Ideal** **Clothing**	16.55 (3.52) 8.39 (4.44) 8.61 (3.59) 13.00 (4.37) 17.24 (4.91) 20.88 (2.91) 13.61 (4.77)		18.33 (3.75) 9.10 (4.94) 7.74 (3.91) 15.89 (4.32) 14.47 (4.34) 19.23 (2.92) 15.41 (4.69)				14.39 (3.95) 8.55 (4.56) 8.16 (3.61) 12.76 (4.46) 15.21 (5.74) 15.89 (4.48) 14.97 (5.36)	17.11 (5.03) 8.66 (4.35) 7.37 (3.43) 16.63 (3.33) 11.77 (4.62) 16.26 (3.93) 18.60 (4.92)		< .001 .879 .526 < .001 < .001 < .001 < .001	

#### 3.2.1 BID sub-analysis within dancer population

To understand the sample further, mean BID scores were calculated for each level of dance, within the wider dancer group (Table 3). A Shapiro-Wilk test of normality was conducted, revealing BID across the three groups to violate normal distribution, p = .011. Therefore, a non-parametric test was ran using an independent samples Kruskal-Wallis. The test revealed that BID significantly differed across the three groups, χ^2^(2, N = 106) = 7.061, p = .029. Multiple pairwise comparisons with a Bonferroni corrected significance level, however, did not reveal any significant differences.

**Table 3:** BID of dancer population N = 106.

	Amateur (N = 56)	Pre-professional (N = 36)	Professional (N = 14)	
	Mean (SD)	Mean (SD)	Mean (SD)	p
**Body Image Dissatisfaction (BID)**	47.71 (15.23)	55.03 (15.19)	43.63 (16.22)	.029

As discussed previously, BID is common among ballet dancers, therefore, the dancer group was split into those with ballet training and those without, whereby mean BID scores were calculated (Table 4). Independent samples t-test revealed no significant difference in BID score between dancers with ballet training and other genre dancers.

**Table 4:** BID of dancers with ballet vs no ballet training N = 106.

	Ballet Training (N = 72)	Other Genre Dancers (N = 34)
	Mean (SD)	Mean (SD)
**Body Image Dissatisfaction (BID)**	49.31 (16.51)	50.32 (15.80)

### 3.3 Bivariate correlations

Bivariate correlations were calculated between all variables using an alpha significance level of .05 (Table 5). In line with Pearson’s r guidelines [65] all predictors, apart from muscular ideal, displayed medium to large significant positive correlations with BID, whereby clothing demonstrated the largest (*r* = .685, *p* < .001), followed by the thin ideal (*r* = .625, *p* < .001), media (*r* = .624, *p* < .001), family (*r* = .409, *p* < .001), athletic ideal (*r* = .315, *p* < .001) and finally peers (*r* = .305, *p* < .001). There were small to large significant correlations between family and peers (*r* = .504, *p* < .001), family and media (*r* = .339, *p* < .001) and peers and media (*r* = .270, *p* < .001) unsurprising given TIM. Large significant correlations were seen between the thin and athletic ideal (*r* = .616, *p* < .001) and between the thin ideal and media (*r* = .505, *p* < .001), again unsurprising given the mediating role of ideal internalisation within TIM. Significant correlations were also seen between thin ideal and clothing (*r* = .390, *p* < .001), thin ideal and peers (*r* = 281, *p* < .001) and lastly between thin ideal and family (*r* = .230, *p* = .002). Further significant correlations were found between athletic and muscular ideals *(r* = .480, *p* < .001), athletic ideal and peers (*r* = .270, *p* < .001) and the athletic ideal and media (*r* = .186, *p* = .013). Finally, clothing significantly correlated with all tripartite influences, media (*r* = .564, *p* < .001), family (*r* = .366, *p* < .001) and peers (*r* = .311, *p* < .001).

**Table 5:** Bivariate correlation matrix for body dissatisfaction, thin ideal, family, peers, media, muscular ideal, athletic ideal and clothing scores. N = 179.

	1	2	3	4	5	6	7	8
**1. Body Dissatisfaction**	**-**							
**2. Thin Ideal**	.625***	-						
**3. Family**	.409***	.230**	-					
**4. Peers**	.305***	.281***	.504***	-				
**5. Media**	.624***	.505***	.339***	.270***	-			
**6. Muscular Ideal**	.024	.124	.000	.078	-.063	-		
**7. Athletic Ideal**	.315***	.616***	.077	.270***	.186*	.480***	-	
**8. Clothing**	.685***	.390***	.366***	.311***	.564***	-.106	.057	**-**

* Correlation is significant at p < .05.

** Correlation is significant at p < .01.

*** Correlation is significant at p < .001.

### 3.4 Multivariate analyses

Prior to conducting hierarchical multiple regression analysis, relevant assumptions were assessed. Firstly, the sample size of 179 (more than 30 per group) was deemed sufficient for analyses in line with the prospective priori power analysis. Whilst the bivariate correlations (Table 6) confirmed there was no singularity between variables (*r* did not equal ± 1), they did reveal significant correlations between some variables. The assumption of no multicollinearity was met despite significant correlations; all correlations were less than 0.8.

**Table 6:** Hierarchical multiple regression analysis for male non-dancers. N = 38.

Regression	β	R^2^	ΔR^2^	ΔF
**Block 1** Age BMI	-.033 .257	.064	.064	1.19
**Block 2** Age BMI Thin Ideal Family Peers Media Muscular Ideal Athletic Ideal Clothing	-.140 .084 .174 .041 -.199 .269* .083 .134 .564***	.742	.679	10.54***

* Significant at p < .05.

** Significant at p < .01.

*** Significant at p < .001.

#### 3.4.1 Hierarchical multiple linear regression analyses

Hierarchical multiple regression analysis was used to assess the extent to which BID could be uniquely predicted by the thin, muscular and athletic ideals, family, media, and peer pressures and clothing practices after controlling for age and BMI. Subsequently, the individual predictor contribution was assessed.

For male non-dancers (Table 6), hierarchical multiple regression revealed that block 1 (B1) accounted for 6.4% (R^2^ = .064) of the variance in BID scores. Block 2 (B2) was able to account for 74.2% (R^2^ = .742) of the variance in BID scores. This .679 change in R^2^ was significant [ΔR^2^ = .679, *F*(2,35) = 8.96, *p* < .001], revealing that the predictors under investigation were able to uniquely predict BID within male non-dancers over and above age and BMI. Individual predictor contribution revealed significant contributions from clothing [β = .564, *t*(37) = 3.63, *p* < .001] and media [β = .269, *t*(37) = 2.07, *p* = .048].

For male dancers (Table 7), hierarchical multiple linear regression revealed that B1 accounted for 6.5% (R^2^ = .065) of the variance in BID score, whilst B2 accounted for 73.4% (R^2^ = .734) of the variance in BID scores. This .670 change in R^2^ was significant [ΔR^2^ = .670, *F*(2,32) = 8.27, *p* < .001], revealing that the factors in question uniquely predicted the BID of male dancers over and above age and BMI. Individual contribution revealed clothing to be the only significant contributing predictor to the significant model [β = .791, *t*(32) = 4.83, *p* < .001].

**Table 7:** Hierarchical multiple regression analysis for male dancers. N = 33.

Regression	β	R^2^	ΔR^2^	ΔF
**Block 1** Age BMI	-.166 .230	.065	.065	1.04
**Block 2** Age BMI Thin Ideal Family Peers Media Muscular Ideal Athletic Ideal Clothing	-.221 .363 .012 .013 -.022 -.061 .146 .297 .791***	.734	.670	8.27***

* Significant at p < .05.

** Significant at p < .01.

*** Significant at p < .001.

For female non-dancers (Table 8), hierarchical multiple linear regression revealed that B1 accounted for 15.4% (R^2^ = .154) of the variance in BID score, whilst B2 accounted for 75.2% (R^2^ = .752) of the variance in BID scores. This .598 change in R^2^ was significant [ΔR^2^ = .598, *F*(2, 32) = 8.60, *p* < .001], again revealing that the investigated factors uniquely predicted BID of female non-dancers over and above age and BMI. Individual contribution revealed significant predictor input from media [β = .303, *t*(34) = 2.12, *p* = .045] and clothing [β = .316, *t*(34) = 2.26, *p* = .033].

**Table 8:** Hierarchical multiple regression analysis for female non-dancers. N = 35.

Regression	β	R^2^	ΔR^2^	ΔF
**Block 1** Age BMI	.085 .346	.145	.145	2.91
**Block 2** Age BMI Thin Ideal Family Peers Media Muscular Ideal Athletic Ideal Clothing	.086 .127 .024 .208 -.031 .303* .255 -.258 .316*	.752	.598	8.60***

* Significant at p < .05.

** Significant at p < .01.

*** Significant at p < .001.

Finally, for female dancers (Table 9), hierarchical multiple linear regression revealed that B1 accounted for 7.8% (R^2^ = .078) of the variance in BID score. B2 accounted for 71.0% (R^2^ = .710) of the variance in BID scores. This .632 change in R^2^ was significant [ΔR^2^ = .632, *F*(2, 70) = 19.59, *p* < .001], revealing that all seven factors investigated uniquely predicted the BID of female dancers over and above age and BMI. Individual significant predictor contributions highlighted the thin ideal [β = .531, *t*(72) = 5.42, *p* < .001] family [β = .187, *t*(72) = 2.26, *p* = .027] and clothing [β = .245, *t*(72) = 2.88, *p* = .005].

**Table 9:** Hierarchical multiple regression analysis for female dancers. N = 73.

Regression	β	R^2^	ΔR^2^	ΔF
**Step 1** Age BMI	-.251 .104	.078	.078	2.96
**Step 2** Age BMI Thin Ideal Family Peers Media Muscular Ideal Athletic Ideal Clothing	-.076 .045 .531*** .187* -.077 .150 .148 -.035 .245**	.710	.632	19.59***

* Significant at p < .05.

** Significant at p < .01.

*** Significant at p < .001.

### 3.5 Summary of findings

The main findings revealed that all seven factors under investigation uniquely predicted BID, above age and BMI, within all four groups. Regarding individual predictor contribution, clothing provided the only significant result within all four groups.

## 4. Discussion

This study investigated seven predictors of body image dissatisfaction (BID), aiming to identify differences between four independent groups (male dancers, female dancers, male non-dancers, female non-dancers). It was predicted that greater media pressure would significantly predict higher BID scores over and above other factors in male and female non-dancer categories. It was predicted that greater levels of discomfort in tight-fitting clothing would significantly predict higher BID scores over and above other factors, among female dancers. It was predicted that the greater internalisation of the athletic ideal would significantly predict higher BID scores over and above other factors, among male dancers. Finally, it was predicted that the remaining factors would follow a similar trajectory whereby greater internalisation/pressures would significantly predict higher BID. Whilst the first hypothesis was fully met, the following hypotheses were only partially met. This will be discussed in the following.

Whist there is wealth of existing literature suggesting that age and body mass index (BMI) play a large part for body dissatisfaction [66–68], the current study did not find these factors as meaningful predictors for body dissatisfaction. It must be acknowledged that the current study was not set to investigate the impact of age and BMI, and the recruitment strategy was not addressing these, which might explain the lack of contribution of such factors. In the following it must be acknowledged that the participant sample is representing young adults with mostly healthy weight and thus the results may not be necessarily applicable to a wider participant group.

The seven predictors of interest together significantly accounted for variance in BID within all four participant groups; variances of 73.4%, 71.0%, 74.2% and 75.2% were significantly accounted for within male dancers, female dancers, male non-dancers and female non-dancers respectively. When assessing individual predictor contributions, clothing was identified as the only factor that predicted BID in all four participant groups. Indeed, for male dancers, the clothing factor was the only factor that predicted BID when all seven factors were entered in the model simultaneously. Within female dancers, internalisation of the thin-ideal and family pressures significantly contributed alongside clothing. As for male and female non -dancers media pressure significantly contributed alongside clothing. The significance of clothing as a predictor of BID within all four groups is an interesting finding and will be discussed, alongside other results.

Clothing practices, specifically discomfort in tight-fitting garments during physical activity, was the only significant predictor of BID within all four groups and is a novel finding. Previous literature has highlighted only associations between clothing and BID which predominantly focused on female dancers [18,36] Other than previously mentioned correlational findings, there is very little research on clothing and its impact on body satisfaction. To the authors knowledge the only experimental finding is by [69] who assessed an intervention for female ballet dancers, which introduced leotard free classes, and found a reduction in disordered eating behaviours following the intervention. Within the female dance world, strict implementation of tight clothing is viewed as vital to ensure correct posture, however, subsequent attention can then be brought to the dancer’s perceived bodily flaws [37] providing a possible explanation for the predictive ability clothing possessed for female dancers. The current study used a sample of dancers engaged in genres other than ballet, suggesting attention brought to one’s bodily flaws possibly facilitated by tight-fitting clothing is apparent across many dance styles, not just ballet. Whilst the finding was not surprising with regards female dancers, it was interesting that the clothing predicted body dissatisfaction in all participant groups.

Clothing significantly contributed to the prediction of BID within male dancers, which proved difficult to evaluate due to the lack of male focused research. Nevertheless, this result could be considered unsurprising given male dancers are argued to face scrutiny [70] and measurement against idealised body types [71] in much the same way as their female counterparts. Like female dancers, males are expected to display their physique through tight clothing as stated in the well-respected International Dance Techers Association syllabus [72]. Perhaps a similar critical gaze is placed upon the male dancer’s body, possibly explaining their elevated levels of BID predicted by the enforcement of tight-fitting dance clothing.

Literature for non-dancing males is similarly scarce. Nonetheless, this study’s findings are in line with the limited available research; [73] found millennial males tended to wear clothing to conceal their body when reporting unhappiness toward their body image. Similarly, the findings by [74] suggest that among the general population, male BID is positively related to concerns surrounding the fit of and overall bodily appearance in clothing. Moreover, this study’s findings found clothing to predict female non-dancer’s BID which is consistent with literature depicting clothing-related appearance management as associated with increased BID and perceived body image as related to clothing fit [63,75]. Given the findings within the general female and male population, it is perhaps unsurprising that clothing acted as a predictor of BID for these groups. In light of current and previous findings, clothing practices influencing male and female BID appear widespread, perhaps as clothing is a highly accessible means to avoid physical appearance when dissatisfied [76] and exacerbates dissatisfaction when tight and clinging to unfavourable parts of the individual’s body, possibly evoking thoughts surrounding weight gain [77–79]. It should be noted the explanation of such a result is speculative given there is a scarcity of research surrounding clothing and body image, highlighting the need for further investigation.

Further findings revealed that media pressure, out of the three components of Tripartite Influence Model (family, peers and media) [7], predicted BID among non-dancer groups only. In recent decades, literature has demonstrated the dominance of the media and how advertising unrealistic body standards can have detrimental effects on viewers [80–82]. Media pressures predicting BID in both male and female non-dancers was an expected finding; social media is a highly engaging platform [83] where young adults (aged 18–30) spend an increasing amount of time [84,85]. Within the current study, the average age was 27.6 and 27.3 for non-dancing males and females respectively. The demographic represented in this study, developed during the exponential growth of social media [86,87] which saw users frequently interacting with the media’s projection of unrealistic body standards [88]. It is therefore unsurprising that the media acted as a predictor of BID in this study.

Interestingly, media did not contribute to the prediction of BID within either dancer group, which does not align with previous findings for female dancers reporting BID as predicted by media pressures [34]. One possible explanation could stem from the typical overreliance on female adolescent participants within BID research; the current sample excluded individuals under age 18, suggesting that predictors of adult female dancer’s BID differ to that seen among adolescents. Moreover, Office for National Statistics [89] suggests 89.6% of 16–24-year-olds are in full time education or employment. When paired with demographic information, highlighting hours of dance per week as approximately 7.5 and 19.5 for female and male dancers respectively, this perhaps leaves little free time for exposure to media ideals. Given it has been evidenced that higher frequency of use/ more time spent on social media is associated with body dissatisfaction among young adult samples [90,91] but with no evidence that intensity of the engagement in social media content predicts the BID, it was speculated that the non-dancer group with intrinsically having more time available, might be more influenced. However, it must be acknowledged that this discussion is speculative, and that researchers are proposing this as one possible explanation rather than a conclusive explanation. Alternatively, explanation could stem from social psychology, whereby evidence suggests that belonging to a social group can have beneficial effects on well-being [92]. Perhaps belonging to the social group of dance may explain why media pressures did not predict BID in dancers; belonging within the dance community could act as a protective factor, an interesting prospect to be followed up.

In contrast to female non-dancers, family pressures rather than media pressures significantly contributed to the prediction of BID among female dancers. According to the research by Francisco [93] direct parental influences were a significant predictor of BID among female aesthetic athletes, more specifically critical comments made surrounding weight. Similarly, Doria and Numer [94] highlighted indirect parental comments influenced the relationship female dancers had with their bodies, particularly comments about other dancers’ bodies, intensifying participants’ insecurities. Possible explanations for the current results may be drawn from such research; parental comments surrounding body image (directly and indirectly) could induce a sense of BID among female dancers. Alternatively, males typically receive less family support within dance [43,95,96] providing possible explanation why family pressure predicted BID within female but not male dancers.

Another significantly contributing predictor for female dancers was internalisation of the thin ideal, which is unsurprising considering the relentless quest to achieve the perfectly lean body inherent within female dance [23,33,97] Previous research indeed indicates an association between the drive for thinness and BID [21,22,98] Among female non-dancers thin ideal internalisation had no significant predictive contribution to BID. Furthermore, ideal internalisation was not found at any individual contributor capacity within male groups which could indicate that different populations have different tendencies to internalise idealised bodies, for example, from this research it would appear females, namely dancers, possess this tendency.

Internalisation of the thin ideal within the female dancer group could stem from an overrepresentation of ballet within the sample. Although past research was expanded on through inclusion of multiple genres, almost 50% of the female dancer sample had ballet training whereas the figure was 30% in males. Given the widely evidenced association between drive for thinness and ballet [24,99] this could explain why the thin ideal was a predictor only for female dancers. Overrepresentation of ballet is perhaps difficult to avoid; ballet provides a strong technical base, required for all dance styles, and usually precedes training in other styles [100]. For instance, Weiss et al. [101] revealed female dancers typically started their training with ballet and although some male dancers followed the same trajectory, they predominantly started with jazz.

Male dancers appeared influenced only by clothing within this study; clothing practice was the only significantly contributing predictor of BID within this group. As mentioned above this is an interesting result given, to the best of the researcher’s knowledge, it has not been previously demonstrated. Explanations may stem from the large number of professionals in the sample compared to the female dancers. Professional dancers are more established within dance compared to other levels, whereby sharing their achievements can make aspiring dancers feel intimidated and discouraged [102]. The physically demanding job of professional dancers is well documented, the schedule of which often limits their time for social involvement [103–105] The lack of time to be present on social media and at family/peer activities, perhaps reduces exposure to scenarios that could be influential on body image and dissatisfaction, possibly explaining the lack of significant predictor contributions seen for the male dancer sample; they were heavily weighted by professionals.

The current findings present significant implications for dancers and non-dancers in terms of BID prevention. Firstly, irrespective of sex or dance status, clothing practices predicted BID across all participants, suggesting that feelings of discomfort in tight-fitting garments is an issue within the general population and at-risk groups e.g., dancers. Clothing practices are highly relevant to an individual’s life and require further investigation as a predictor BID; this area is in preliminary stages among current literature. In terms of dance, the strict implementation of tight-fitting attire for males and females could increase risk of developing of BID, possibly due to all bodily aspects being visible which subsequently can lead to feelings of discomfort. Wherever practical, traditional dance aesthetics that emphasise one’s silhouette whilst performing could be relaxed to lessen possible discomfort and prioritise well-being [18,69].

Similarly, in the general population, reducing the emphasis on tight clothing like that seen within the athleisurewear market, which in recent years has become fashionable street wear [106–108], could assist in normalising looser clothing during physical activity, ensuring individuals are more comfortable and less likely to develop BID. Alternatively, from a clinical perspective, given clothing predicted BID in all groups it may be useful to consider an individual’s clothing practices when assessing BID and/or ED; an individual suffering from BID may choose to camouflage their body, avoiding tight garments.

Further valuable implications surround media pressures predicting BID within male and female non-dancers. It is well documented that in recent years the role of social media has grown exponentially and dominates everyday life. Current results, paired with the collected demographic information, suggest individuals from the general population possibly have more free time than individuals that dance regularly, potentially affording more opportunity for exposure to idealised media content. Considering the findings, it could be useful to encourage preventative measures like smartphone use notifications, to increase awareness of time spent on a given social media platform [109]. By implementing this, individuals in the general population could become more aware of media influences and spend their free time more productively, protecting their bodily related wellbeing.

Whilst the research has numerous strengths, it does not come without limitations. Regarding strengths, arguably, the use of a large participant sample consisting of both males (N = 71) and females (N = 108) is unique to the type of research. The recruitment of male participants (38 non-dancer and 34 dancer) is a particular strength; the current BID literature is lacking in representation of the male demographic, particularly within dance. Furthermore, the study utilised many predictors, typically unseen within the research, which has usually focused on one to three predictors [34,110,111]. Thus, utilising seven predictors alongside many male and female participants adds great strength to the current research.

However, it must be acknowledged that there was possible overrepresentation of ballet within the female dancer sample; almost 50% of female dancers had ballet involvement. Within dance, it is argued that ballet provides technique translating to all other genres, acting as a foundation IDTA [112]. Despite researchers’ best efforts to move the focus away from ballet it was still largely apparent across the female dancers and could have influenced the results, for instance, internalisation of the thin ideal has been consistently linked with female ballet dancers [24] and was a significant predictor in this research.

Further concern relates to possible overrepresentation of professionals within the male dancer sample. Examining differences between the varying levels of dance among participants was beyond the scope of the current research aims but a factor to be investigated, nonetheless. Demographic data gathered revealed the dancer sample ranged from amateurs to professionals, whereby the male dancer sample was heavily weighted by professionals in comparison to the female dancers. Findings may have been impacted by the professional dancer imbalance when considering the different challenges faced by dancers at different levels [113].

Future research would benefit from replicating the current study with a focus on potential differences between each level of dance. Moreover, research could recruit dancers whose main genre is not ballet, for instance an individual who identifies and trains solely as a tap dancer. Alternatively, to truly expand the field away from ballet, research could exclude participants with any involvement in ballet, albeit the sample may be quite small considering how widespread ballet training is.

To conclude, the research revealed that BID can be predicted by clothing within dancer and general populations for both males and females. Moreover, media pressures were highlighted as a predictor of BID within the general population. Both main findings have important implications which apply to males and females. Implications of the findings relate to the relaxation of tight-fitting clothing within the dance environment and general population activewear. Allowing individuals to feel more comfortable during physical activity and general day-to-day life could aid the reduction of BID and the subsequent development into an eating disorder. Overall, the research findings are an invaluable contribution to the body image literature, specifically contributing to the understanding of male body image and hopefully inspiring necessary further study.

### 4.1 Clothing questionnaire

Please rate how much you agree or disagree with the following statements (select only one answer per statement):

**Table:** 

Question					
1. I wear clothing that will divert attention from my weight.	Definitely Disagree	Disagree	Neither Agree nor Disagree	Agree	Definitely Agree
2. I wear clothing that camouflages my body shape	Definitely Disagree	Disagree	Neither Agree nor Disagree	Agree	Definitely Agree
3. I feel comfortable about my bodily appearance in revealing clothing during physical activity	Definitely Disagree	Disagree	Neither Agree nor Disagree	Agree	Definitely Agree
4. Wearing tight-fitting clothing makes me feel uncomfortable about my bodily appearance	Definitely Disagree	Disagree	Neither Agree nor Disagree	Agree	Definitely Agree
5. I wear baggy clothing during physical activity	Definitely Disagree	Disagree	Neither Agree nor Disagree	Agree	Definitely Agree

## Supporting information

S1 File In-house created questions. (DOCX)
