# Supplementary material for: The ‘ideal’ dancer: An investigation into predictors of body image dissatisfaction among male dancers, female dancers and their non-dancing counterparts
Source: PLoS One. 2024 Nov 7;19(11):e0313142. doi: 10.1371/journal.pone.0313142 (PMC11542894; doi:10.1371/journal.pone.0313142)
Supplement: S1 File — (DOCX) [file pone.0313142.s001.docx]

**CLOTHING**

Q1. I wear clothing that will divert attention from my weight.

Q2. I wear clothing that camouflages my body shape

Q3. I feel comfortable about my bodily appearance in revealing clothing during physical activity

Q4. Wearing tight-fitting clothing makes me feel uncomfortable about my bodily appearance

Q5. I wear baggy clothing during physical activity

**ATHLETIC IDEAL**

Q1. It is important for me to look athletic

Q2. I spend a lot of my time doing things to look more athletic

Q3. I think a lot about looking athletic

Q4. I want my body to appear lean with little body fat

Q5. I aspire to look slim and toned

**MUSCULAR IDEAL**

Q1. I think a lot about looking muscular

Q2. I spend a lot of time doing things to look more muscular

Q3. It is important for me to look muscular

Q4. I find myself frequently exercising to gain muscle

Q5. I aspire to “bulk up” and look muscular
